# Further characterization of Tsol-p27 as a diagnostic antigen in sub-Saharan Africa

**DOI:** 10.1016/j.exppara.2013.09.006

**Published:** 2013-09-13

**Authors:** Noémia Nhancupe, Fernando Salazar-Anton, Emília Virginia Noormahomed, Sónia Afonso, Johan Lindh

**Affiliations:** a Departamento de Microbiologia, Faculdade de Medicina, Universidade Eduardo Mondlane, Mozambique; b Microbiology, Tumor and Cell Biology, Karolinska Institutet, Sweden; c Department of Microbiology and Parasitology, Faculty of Medical Sciences, National Autonomous University of Nicaragua, León, Nicaragua; d Department of Medicine, University of California, San Diego, CA, USA; e Departamento de Paraclínicas, Faculdade de Veterinária, Universidade Eduardo Mondlane, Mozambique; f Epidemiology and Evaluation, Swedish Institute for Communicable Disease Control, SE17182 Solna, Sweden

**Keywords:** Cysticercosis, Diagnosis, Immunoreactive proteins, Serology, *Taenia solium*

## Abstract

Commercial antigens used to diagnose human neurocysticercosis are obtained from either a soluble parasite extract or a parasite-derived glycoprotein fraction. The aim of the present study was to identify antigenic proteins as potential diagnostic candidates in Mozambique. Soluble proteins from *Taenia solium* cysticerci were separated by two-dimensional electrophoresis and blotted onto nitrocellulose membranes. Subtracted hybridization was performed with serum samples obtained from patients with neurocysticercosis (NCC) and from a NCC-negative control group. Six antigenic proteins were identified and sequenced by liquid chromatography–mass spectrometry. Among these we found Tsol-p27, which was previously identified as a diagnostic candidate in a study conducted in Nicaragua, Central America. Here, we evaluated Tsol-p27 and the antigen cC1 as potential recombinant diagnostic reagents, and also investigated the localization and partial function of Tsol-p27. Immunoblotting demonstrated that Tsol-p27 was recognized by all 10 serum samples from NCC-positive individuals, whereas cC1 was identified by only five of the 10 positive sera. None of the antigens were recognized by negative control sera. Despite the limited number of serum samples evaluated in this study, the results suggest that Tsol-p27 can be a suitable candidate for diagnosis of human NCC, not only in Central America but also in sub-Saharan Africa.

## 1. Introduction

The larval form of *Taenia solium* is called *Cysticercus cellulosae*, and it is a parasite that causes cysticercosis, a neglected and potentially eradicable disease in both humans and pigs (Mwanjali et al., 2013). Humans serve as the definitive host, where infection in the intestine is termed taeniasis. Pigs are the intermediate host, and they become infected through ingestion of human faeces in environments with poor sanitation. Once in the stomach of a porcine host, an egg hatches to release the larval stage, which migrates to the muscles or the brain where it becomes encysted. Humans are infected when they unintentionally ingest *T. solium* eggs that are present in food or water that has been contaminated with human faeces (Garcia and Del Brutto, 2000). If *T. solium* larvae reach the central nervous system in humans, they can cause neurocysticercosis (NCC), which is the most serious manifestation of infection with this parasite. NCC is a major cause of adult-onset epilepsy in areas where the disease is endemic, and it has been estimated that approximately 1.7–3.0 million people worldwide suffer from such epilepsy (Nash and Garcia, 2011). Furthermore, NCC is the most common cause of epilepsy in children and should be suspected in paediatric patients presenting with convulsions without fever (Bern et al., 1999; Correa et al., 1999; Fisher et al., 2005).

Little is known about the impact and extent of cysticercosis and NCC in Mozambique and other parts of the world, and this situation is due to a lack of both epidemiological surveys and diagnostic methods that are simple to use, inexpensive, specific, and sensitive. Serological studies of human subjects in Mozambique have indicated that 15–21% of healthy adults are positive for cysticercosis antibodies or antigen, and that seroprevalence is as high as 51% among neuropsychiatric patients (Afonso et al., 2011).

At present, diagnosis of cysticercosis is a complex process based on clinical neuroimaging methods and epidemiological data. The ‘gold standard’ technique is magnetic resonance imaging (MRI), which unfortunately is too expensive to use on the general population and is not available in most hospitals in endemic countries. The most specific test available is an enzyme-linked immunoelectrotransfer blot (EITB) assay based on seven cysticercus glycoproteins purified by lentil-lectin affinity chromatography. This EITB technique has been reported to offer close to 100% specificity and a sensitivity varying from approximately 70% to 90% (Tsang et al., 1989), although one study indicated a sensitivity of only 28% in cases involving single enhancing parenchymal cysts in the brain (Wilson et al., 1991). Several investigators have purified glycoproteins by lentil-lectin affinity chromatography and found that seven bands around 15 to 30 kDa were highly specific to neurocysticercosis (Parkhouse and Harrison, 1987; Tsang et al., 1989). However, these glycoproteins prepared by lentil-lectin affinity chromatography showed cross-reactivity when used as antigens in enzyme-linked immunosorbent assay (ELISA) (Ito et al., 1998). Recently, we developed a simple method to purify diagnostic antigens under non-reducing conditions by preparative two-dimensional electrophoresis (2-DE) from cyst fluid available for both ELISA and immunoblot analysis, and we demonstrated the sensitivity and specificity of this technique for differential serodiagnosis of NCC in Nicaragua (Salazar-Anton and Lindh, 2011; Salazar-Anton et al., 2012). Recombinant antigens have been tested because of their potential for diagnosing this disease, and this group of proteins includes recombinant Tsol-p27, which has been proven useful for such diagnosis in Central America (Salazar-Anton et al., 2012). Despite those findings, no information has been published regarding what antigens might be used in sub-Saharan Africa, nor has it been shown where the potential diagnostic antigen Tsol-p27.localizes localizes in the parasite or what function this protein might have. Therefore, to describe such immunogenic proteins in Mozambique, we performed 2-DE Western blot analysis on NCC-positive and NCC-negative serum samples and tested proteins for their immunogenicity.

Here, we describe the method we used to isolate and express the cC1 and Tsol-p-27 proteins, and also present a further characterization of Tsol-p27 and its value for serodiagnosis of human cysticercosis in Mozambique.

## 2. Materials and methods

### 2.1. Source of antigen

Intact *T. solium* cysts used for determination of immunogenic proteins were obtained from naturally infected pigs from an endemic area of Mozambique. The cysts were washed with phosphate-buffered saline (PBS; pH 7.5) and kept at −80 °C until used. Briefly, cysticerci were mechanically disrupted in 500 μl of PBS and homogenized with a protease inhibitor cocktail (Invitrogen^®^). The preparation was centrifuged at 13,000*g* for 10 min at 4 °C, and the supernatant was stored at −20 °C until used.

### 2.2. Source of human sera

One serum sample was obtained from each of 20 epileptic patients who who lived in Tete Province in Mozambique and were found to be positive or negative for NCC by Ag ELISA. The patients were also examined individually by computed tomography, (CT) and, based on the results of both tests, their serum samples were divided into a NCC-positive group (*n* = 10) and a NCC-negative control group (*n* = 10) (Afonso et al., 2011; Pondja et al., 2010).

### 2.3. Two-dimensional electrophoresis (2-DE)

IPG strips with a linear pH range of 4–10 or 4–7 (GE Healthcare) were rehydrated for 12 h according to manufacturer’s protocol. The extracted proteins were mixed with rehydration buffer containing 6 M urea, 2 M thiourea, 2% (w/v) CHAPS, 0.4% (w/v) DTT, and 0.002% (w/v) bromophenol blue. The isoelectric focusing (IEF) was performed using a Multiphor system (Pharmacia Biotech) and the following conditions: 200 V for 20 min, 450 V for 15 min, 750 V for 15 min, and 2000 V for 30 min. In the second dimension, the strips from IEF were loaded for sodium dodecyl sulphate poly-acrylamide gel electrophoresis (SDS–PAGE; 12%) according to the protocol provided by the manufacturer (Pharmacia Biotech).

### 2.4. Western blot analysis and protein sequencing

After 2-DE, the acrylamide gels were directly stained with Coomassie Blue (Bio Rad) or transferred to nitrocellulose membrane (GE Healthcare). Membranes blotted with proteins separated by 2-DE or proteins blotted with recombinant cC1 or recombinant Tsol-27 antigens were blocked for 2 h with 5% skim milk in PBS (blocking solution) and subsequently rinsed in PBS and 0.05% Tween 20 (washing solution). Thereafter, the membranes were incubated for 3 h with human sera diluted 1:500 in blocking solution and then for 1 h with rabbit anti-human IgG conjugated with peroxidase (Sigma) diluted 1:3000 in blocking solution. Detection was performed according to the ECL plus manual (GE Healthcare).

The proteins from the blot that reacted with positive sera were identified by comparing with Coomassie stained gel. The protein spots exhibiting the greatest immunoreactivity were selected and excised from the gel and stored at −20 °C. The excised spots were trypsinized, and the proteins were amino acid sequenced at the Protein Analysis Center, Karolinska Institutet, Sweden, by liquid chromatography-mass spectrometry LC–MS).

### 2.5. Cloning, expression, and purification of recombinant proteins

Recombinant Tsol-p27 and cC1 proteins were produced from selected cDNA and amplified using specific primers. For cC1, we used the primer pair cC1F 5′-CTC GGA TCC ATG GCC TAC TGT CGC TCC CTG-3′ (sense)/cC1 R 5′-CGG GAA TTC CGG TGC AGG GCC GAT GAG TTT CA-3′ (antisense). The Tsol-p27 protein was expressed as previously described (Salazar-Anton and Lindh, 2011). All polymerase chain reaction (PCR) experiments were performed using a Thermo Hybrid system and Maxima Hot Start PCR 2xMaster Mix (Fermentas). The PCR conditions were as follows: one cycle at 96 °C for 5 min followed by 30 cycles at 96 °C for 45 s, 52 °C for 45 s, 72 °C for 1 min, and 72 °C for 7 min. The cC1-amplified ORF was cloned into *pTrcHis2* C (Life Technologies) and expressed as a recombinant protein. The recombinant cC1was purified using his selected nickel magnetic beads (Sigma). The resin was equilibrated with a buffer containing 50 mM sodium phosphate (pH 8.0), 0.3 M sodium chloride, and 10 mM imidazole, and then eluted with a higher concentration of imidazole (250 mM). The histidine-containing proteins were separated from the fraction by applying a magnetic separator to the mixed solution. The Tsol-p27 was purified in parallel as described previously (Salazar-Anton and Lindh, 2011).

### 2.6. Polyclonal sera produced in rabbits

The recombinant Tsol-p27 protein was expressed as glutathione S-transferase (GST) fusion protein and then purified as previously described (Salazar-Anton and Lindh, 2011). The purified Tsol-p27 protein was used to raise polyclonal antibodies in rabbits as previously described (Kuratli et al., 2001). In short, on days 0, 28, 56, and 84, each of two male rabbits received four immunizations with the recombinant proteins (250 μg/rabbit) emulsified in 250 μl of Freund’s complete (first immunization) or incomplete (second to fourth immunization) adjuvant. Sera collected before immunizations were used as negative control. Serum was separated and stored at −20 °C until used. All the experiments were carried out with appropriate ethical permission and animals were treated according to the rules of Karolinska Institutet. Animal research is strictly regulated and comes under both Swedish and EU legislation on animal welfare.

### 2.7. Western blot analysis

Western blot analysis of anti-rabbit Tsol-p27 immune sera was performed as in our previous study (Salazar-Anton and Lindh, 2011), with the following changes: extracts from adult tapeworms and cysticerci were separated by 10% SDS–PAGE and transferred to nitrocellulose membranes (Sigma). The blots were washed and incubated for 1 h with the anti-rabbit IgG conjugated with peroxidase (Sigma) and diluted 1:6000 in 5% skim milk-PBS. After washing, the membranes were stained with *3,3*′*,5,5*′*-tetramethylbenzidine* (TMB; Sigma), which is specific for the presence of peroxidase.

### 2.8. Immunohistochemistry

The anti-rabbit Tsol-p27 immune sera were also used to localize the Tsol-p27 proteins in *T. solium* cysticerci. The cysticerci used in this study were dissected from the muscles of a naturally infected pig and then washed in PBS (pH 7.5). Only cysticerci that exhibited a vesicular appearance were selected for the study, and they were stored at −20 °C until used. Ribbons of sections (8 μm) of the *T. solium* cysticerci were placed on slides, and then treated as follows: air dried for 20 min, fixed in acetone for 20 min, and dried for 15 min at room temperature, and thereafter rehydrated in PBS. Anti-rabbit Tsol-p27 or TsolHSP36 immune serum diluted 1:40 in PBS was added to the slides, which were subsequently incubated for 45 min. As a negative control, pre-immune sera were used at the same dilution and incubation time. After the incubation, the slides were washed 2 × 15 min in PBS and then incubated for 45 min with fluorescein isothiocyanate (FITC)-labelled anti-rabbit IgG (Sigma) diluted 1:40 in Evans blue solution. Lastly, the slides were washed 2 × 15 min and examined in a fluorescence microscope (Leica Microsystems).

### 2.9. Amino acid sequencing analysis

Sequencing analysis of Tsol-p27 was performed at the National Center for Biotechnology Information (NCBI; Bethesda, MD, USA) using the BLAST search option with a cut off of 10^−5^. To obtain the family domain description corresponding to Tsol-p27, a cutoff of 10^−9^ was employed in the search option.

### 2.10. Ethical considerations

The investigation of human subjects was approved by the Ethics Committee of the Faculty of Medicine of Eduardo Mondlane University (UEM), the National Committee on Biomedical Research Ethics Ministry of Health of Mozambique, and the Danish National Committee on Biomedical Research Ethics. The production of polyclonal antibodies was approved by the Regional Ethical Committee of Karolinska Institutet, Solna, Sweden (No. N417/10).

## 3. Results

### 3.1. Identification of immunoreactive proteins

**Fig. 1:**
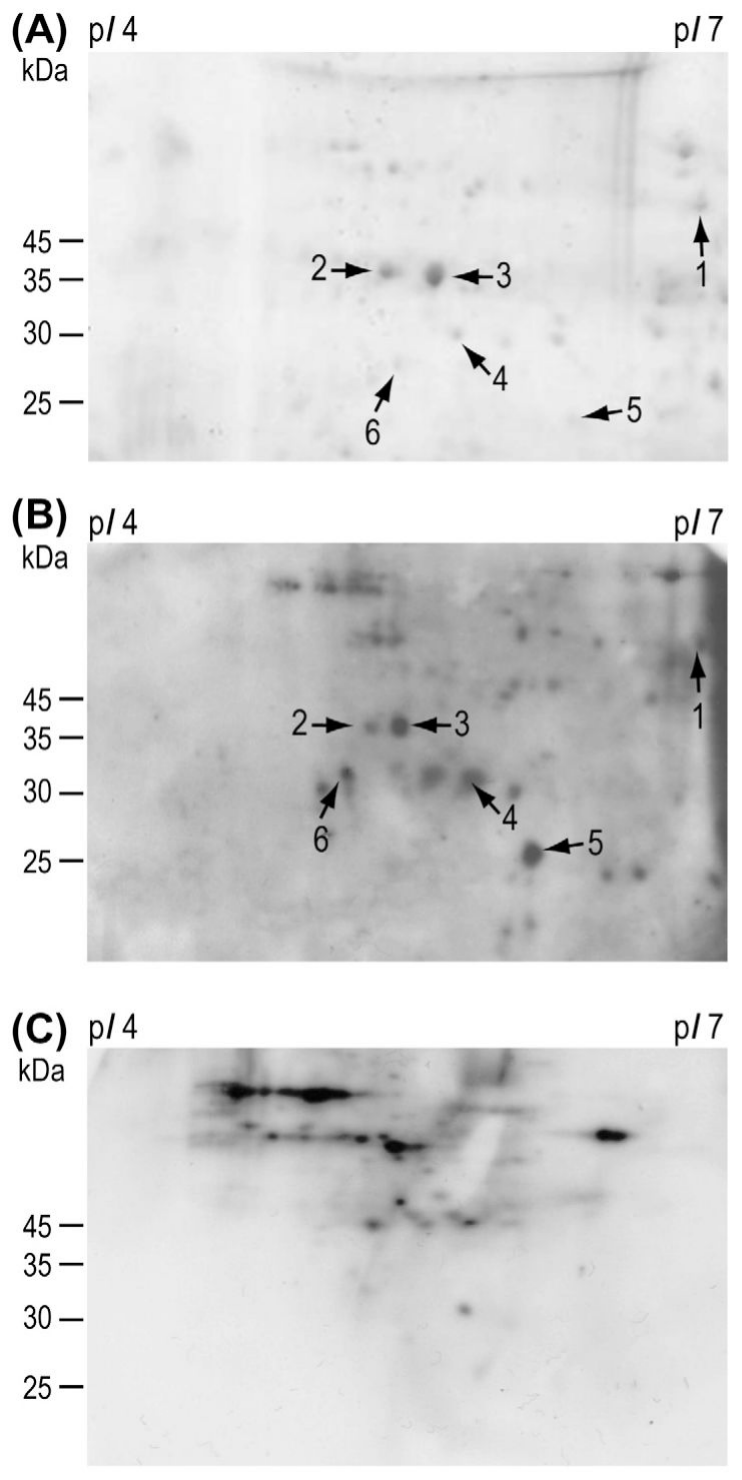
Identification of immunoreactive proteins from *T. solium* cysticerci by 2-DE Western blot analysis. (A) A 2-DE gel stained with Coomassie blue, showing soluble cysticerci proteins with *pI* values in the range 4–7. (B) Corresponding Western blot probed with serum from a patient diagnosed with NCC. (C) Corresponding Western blot probed with serum from a control subject. Arrows indicate the proteins that were isolated and sequenced (Table 1).

The soluble antigens were separated according to their *pI*. Initially, we used a broad *pI* range of 4–10, and, based on those experiments, it could be established that the vast majority of soluble antigens had a *pI* of less than 7, and therefore the remaining experiments were performed at *pI* 4–7. Proteins separated according to their *pI* were followed by molecular weight separation and visualized by Coomassie blue staining (Fig. 1a). The separated proteins were transferred to nitrocellulose membranes and probed four times with NCC-positive and NCC-negative sera, respectively. The proteins that were recognized by sera from patients with NCC (Fig. 1b) but not by sera from the NCC-negative subjects (Fig. 1c) were isolated, which enabled us to localize antigenic spots potentially specific for NCC (Fig. 1a).

### 3.2. Sequencing analysis of six identified antigens

Six spots were cut out from the 2-DE gel and subjected to LC– MS analysis. For four of the six proteins from the gel, the nucleotide sequence could be identified by comparison with four previously reported proteins (Table 1). One of those was Tsol-p27, which was described as a potential diagnostic target molecule in our report from Central America (Salazar-Anton et al., 2012). The remaining two proteins corresponded to tropomyosin and enolase, and could be identified by comparison with published nucleotide sequences from the related *T. asiatica* (ABN14948.1 and ACX56268, respectively).

### 3.3. SDS–PAGE and Western blot analysis of Tsol-p27 and cC1

**Fig. 2:**
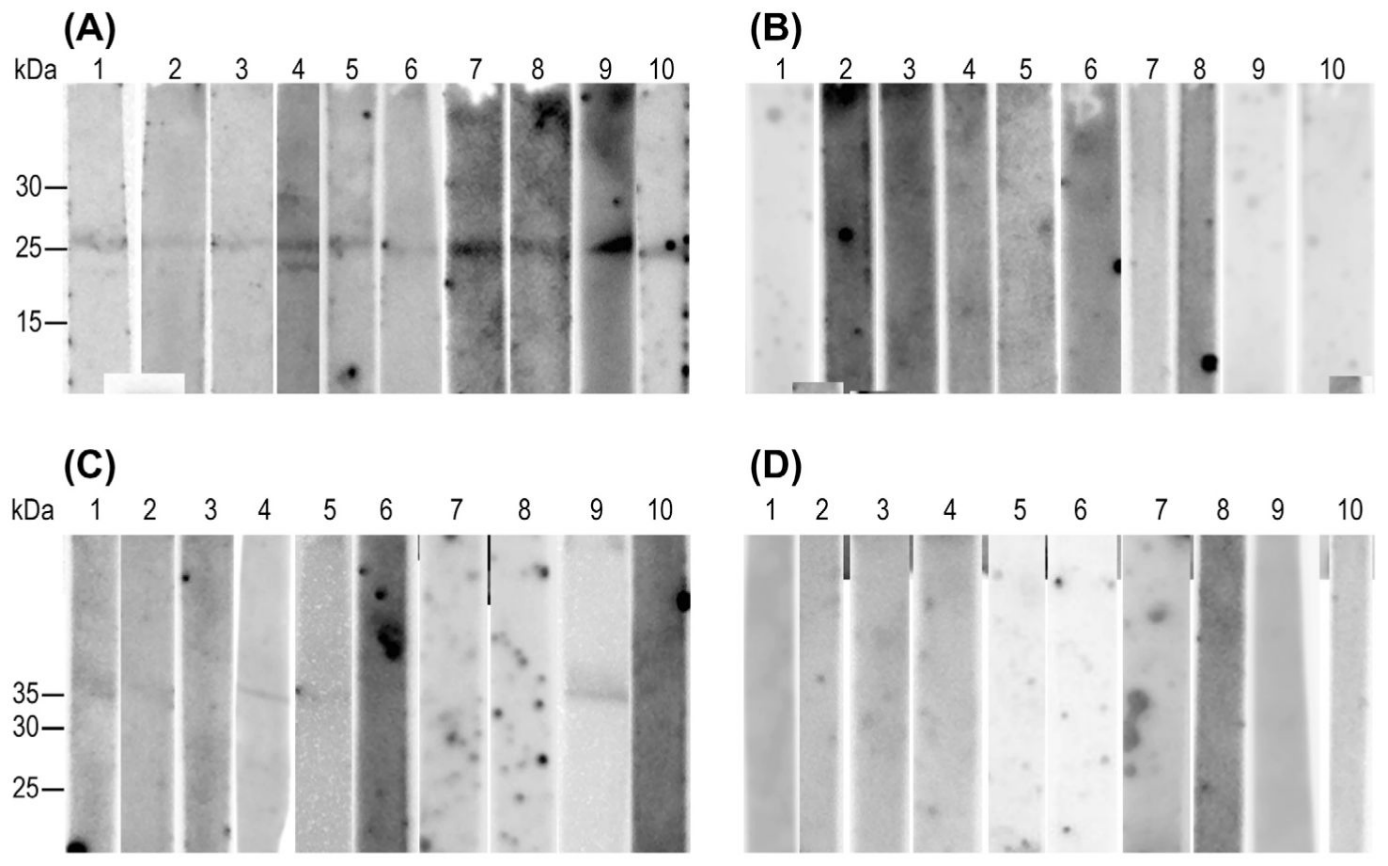
Immunoblot analysis of recombinant antigens Tsol-p27 (A and B) and cC1(C and D). (A and C) Probing done with sera (diluted 1:500) from patients diagnosed as NCC positive by CT (lanes 1–4). (B and D) Probing done with sera (diluted 1:500) from patients diagnosed as NCC negative by CT.

To verify the immunoreactivity of the purified recombinant p27 and cC1, the antigens were transferred to nitrocellulose membranes and probed with the NCC-positive and NCC-negative human sera from Mozambique. Western blot strips of purified Tsol-p27 were recognized by antibodies in all ten of the serum samples in the NCC-positive group (Fig. 2a). In contrast, Western blot strips of purified cC1 were recognized only by antibodies in five of the NCC-positive serum samples (Fig. 2c). None of the antibodies present in the sera from the NCC-negative control group reacted to either the Tsol-p27 or cC1 recombinant antigens (Fig. 2b and d).

### 3.4. Specificity and localization of polyclonal antibodies raised against Tsol-p27

**Fig. 3:**
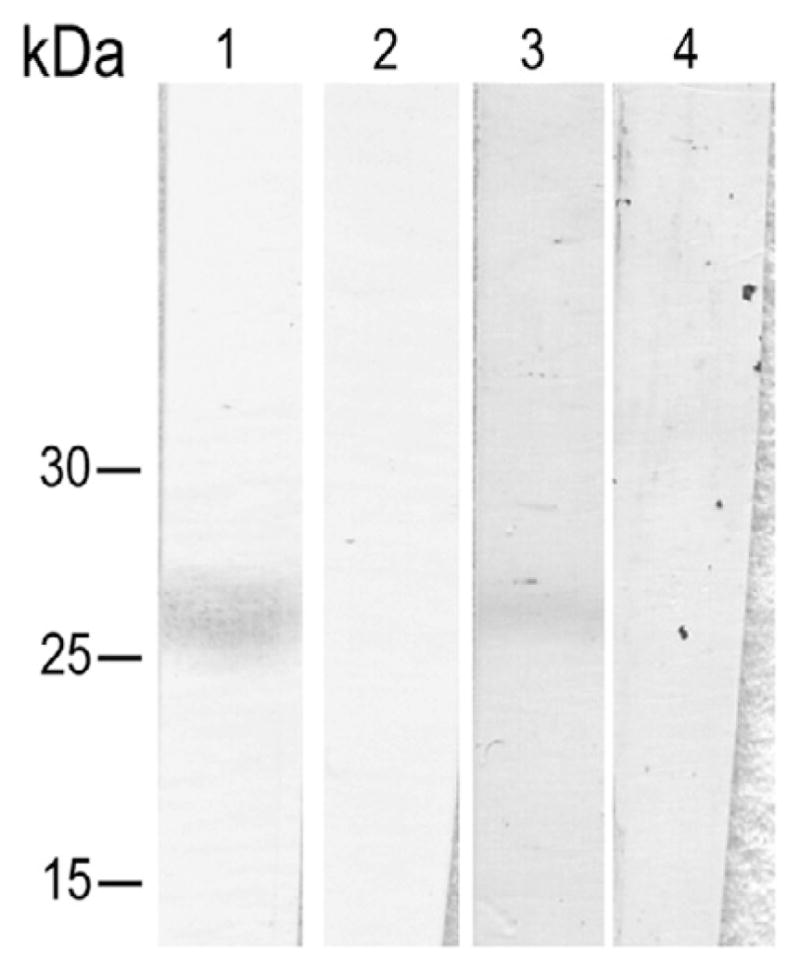
Western blot analysis of extracts of *T. solium* cysticerci (A) and tapeworms (B) using anti-rabbit Tsol-p27 immune sera, and pre-immune sera as negative control Probing was done with Tsol-p27 immune serum in lanes 1 and 3, and with pre-immune serum in lanes 2 and 4.

To determine the specificity of antibodies raised against Tsol-p27 protein, we conducted Western blot analysis using extracts of cysticercus and adult tapeworm. The predicted size of the protein was 27 kDa. Western blotting detected Tsol-p27 protein in both the cysticercus and the adult stage, revealed as a single band of 27 kDa (Fig. 3). Pre-immune serum was used as a negative control (Fig. 3).

**Fig. 4:**
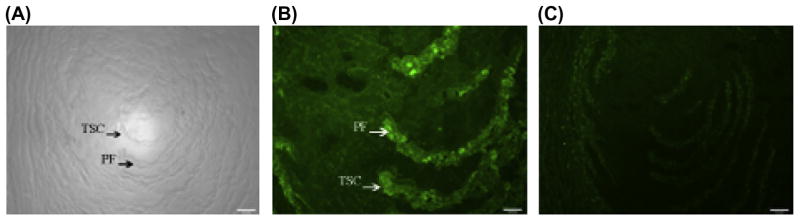
Sections of *Taenia solium* cysticerci examined by phase contrast microscopy and FITC staining. Panels A and B show (arrows) the parenchymal folds (PF) and tegument of the spiral canal (TSC). Panel B is an image obtained using rabbit anti-Tsol-p27 antibodies, and structures are indicated as in panels A and B. Panel C show the results obtained using pre-immune sera as control.

The immunofluorescence reactions of antibodies raised against Tsol-p27 antigen were used to localize the corresponding proteins in *T. solium* cysticercal tissue. The reactions of the antibodies to the Tsol-p27 antigen were seen as intense green fluorescence over the parenchymal folds and the tegument of the spiral canal of *T. solium* cysticerci. No fluorescent reaction was observed when pre-immune serum was tested (Fig. 4).

### 3.5. Sequencing analysis of Tsol-p27

Homologue analysis of Tsol-p27 protein was carried out using BLAST search. The Tsol-p27 amino acid sequence was found to be homologous to the sequences of three immunogenic proteins: P-29 of *Echinococcus granulosus*, as previously described (Salazar-Anton and Lindh, 2011); antigen 6 of *E. multilocularis* (Genbank accession No. AAB61984); antigen 5 of *E. granulosus* (Genbank accession No. ADG37650). Protein family analysis indicated that Tsol-p27 is homologous (*E*-value of <10^−9^) to a group of molecules, cd07594, that include a bin/amphiphysin/rvs (BAR) domain of endophilin-B. The endophilin-B proteins are found in at least 11 different eukaryotic organisms, and they are involved in regulation of intracellular transport, lipid binding, and curvature sensing modules.

## 4. Discussion

Diagnosis of NCC is based on clinical, epidemiological, and laboratory findings, and it can also be accurately achieved using neuroimaging techniques such as CT and MRI (Del Brutto, 1997). Since most of these methods are inaccessible in endemic areas mainly due to cost, other less expensive qualitative techniques are needed (Diaz et al., 1992). Using ELISA, Western blot analysis, or EITB assay to detect specific antibodies against *T. solium* cysticerci has proven to be a useful tool for diagnosing this disease and is therefore also most preferred (Simac et al., 1995). EITB has been reported to offer 98% sensitivity and 100% specificity, although the sensitivity is considerably lower in cases involving single cysts in the brain. This technique requires expensive reagents and advanced equipment, as well as trained staff to carry out the test (Wilson et al., 1991). The antibody-detecting ELISA provides sensitivity and specificity similar to EITB, and use of recombinant antigens in ELISA format has been found to give 90% sensitivity and 100% specificity (Sako et al., 2000). However, both EITB and ELISA results can vary significantly, probably due to the heterogeneity of the patients with respect to their immune status and other aspects, as well as differences in the methods used for antigen preparation. In addition, when crude parasite materials are used as a source of antigens in ELISA, there are a large number of false-positive findings due to cross-reactivity with other parasite antigens (Flisser, 1994).

The aim of the present study was to identify *T. solium* antigenic proteins in Mozambique and evaluate their diagnostic potential in human NCC. The protocol used was similar to earlier published work but with two exceptions (Salazar-Anton and Lindh, 2011): the parasitic material and sera from Mozambique rather than Nicaragua. In general, more antigens were recognized by the control group sera in Mozambique than by the corresponding sera in Nicaragua. This can most likely be explained by a higher background of other parasitic worms infecting the population in Mozambique. However, six different antigens were identified as specific and were detected only in the NCC-positive group. Three of these were revealed to be highly conserved and were also found in other parasitic worms as well as in humans, and hence they were excluded from in further analyses. Two of the antigens, Tsol-p27 and TsolHSP36, were also identified in the group of immune reactive proteins that were detected in Nicaragua (Salazar-Anton and Lindh, 2011). Moreover, extended evaluation of the NCC-positive population studied in Nicaragua had shown the TsolHSP36 to be less sensitive then Tsol-p27 (Salazar-Anton and Lindh, 2011), and thus it was not subjected to further assessment in the present investigation.

Thus, of the six antigens identified in the current study, Tsol-p27 and cC1 were chosen for further analysis by Western blotting. Both these antigens were expressed as recombinant proteins and purified as described in the materials and methods section, and then analyzed by SDS–PAGE. The estimated molecular weight of these two proteins agreed with the molecular weights demonstrated by LC–MS and SDS–PAGE, that is, 27 and 38 kDa for Tsol-p27 and cC1, respectively. The antigen cC1 was recognized by five of the ten NCC-positive serum samples, whereas Tsol-p27 was recognized by all ten of the positive samples. In addition, the bands representing cC1 were generally fainter than the bands for Tsol-p27. The immunoreactivity of cC1 may have been diminished because the recombinant antigen was expressed in *Escherichia coli*, in which glycosylation does not occur. It has been reported that antigens lacking glycosylation exhibit reduced antigenicity (Obregón-Henao et al., 2001).

Several studies, with varying results, have been published that have suggested different antigens as target molecules in serology assays (da Silva et al., 2006; da Silva Ribeiro et al., 2010; Greene et al., 2000; Hancock et al., 2006; Sato et al., 2006; Scheel et al., 2005), and most of those antigens were derived from a family of glycoproteins purified by lentil-lectin affinity chromatography (Tsang et al., 1989). None of those proteins were detected as NCC specific in our settings (Fig. 1), possibly due to disparities between the parasites investigated in different countries with regard to the glycoproteins they expressed (Sato et al., 2006). More precisely, most of the antigens described by other groups were analyzed in South America and Asia (da Silva et al., 2006; da Silva Ribeiro et al., 2010; Greene et al., 2000; Hancock et al., 2006; Sato et al., 2006; Scheel et al., 2005). Despite the limited number of serum samples evaluated in our study, the results suggest that Tsol-p27 can be a suitable candidate for diagnosis of human NCC.

The Tsol-p27 antigen was identified by 2-DE followed by Western blotting and hybridization with human sera confirmed to be positive for NCC in both Central America (Nicaragua) and sub-Saharan Africa (Mozambique). However, localization of Tsol-p27 protein in the *T. solium* cysticercus has thus far been unknown. Here, and Western blot analysis of *T. solium* showed that Tsol-p27 occurs in the adult tapeworm and is also evident in the spiral canal of the cysticercus (Figs. 3 and 4). The presence of this protein in the spiral canal may be related to growth and support of the tapeworm in the human intestine. Furthermore, Tsol-p27 may contribute to attachment of the scolex to the host’s intestinal wall, where it must remain despite the strong peristaltic action of the intestine. We also noted that the tegument of the parasite was reactive to Tsol-p27, which suggests that this protein is involved in the ability of the worm to absorb nutrients from the host (Montero et al., 2007; Rabiela et al., 2000). Sequence analysis of Tsol-p27 protein using a cut-off value of <10^−9^ revealed that it contains an endophilin-B BAR domain. Members of this group of proteins are involved in clathrin-mediated endocytosis, binding to membrane vesicles, and induction of tubular conformation (Farsad et al., 2001). These biological processes are associated with the endoplasmic reticulum, Golgi apparatus, and endocytic vesicles, where they are important for a variety of functions that require membrane sorting, fusion, and transport (Sweitzer and Hinshaw, 1998). It seems that Tsol-p27 plays an essential physiological role in the developmental stages of *T. solium* by regulating clathrin-mediated endocytosis and rearrangement of the cytoskeleton. In addition, it is plausible that Tsol-p27 can participate in the intracellular development of this species by increasing the surface area for exchange between the parasite and its host.

Localization of proteins that are homologues of Tsol-p27 has been determined in various structures in *E. granulosus*. For example, antigen 5 was detected in the germinal membrane, brood capsule, and protoscoleces of that species, suggesting that this protein is associated with degradation of the components of the extracellular matrix that surrounds the parenchyma cells (Sanchez et al., 1993). P-29 is another antigen that is homologous to Tsol-p27, and it has been observed in locations such as the tegumentary syncytium and the rostellum and suckers of protoscoleces (Gonzalez et al., 2000). Those findings may indicate that, similar to Tsolp27, P-29 is involved in the interaction between the parasite and the host mucosal epithelium.

Improvement of immunodiagnostic tests will facilitate a better understanding of the prevalence and epidemiology of NCC in endemic areas (Deckers and Dorny, 2010). Here, we describe a new immunogenic protein, Tsol-p27, which shows promise as a diagnostic antigen for this disease, used alone or in combination with TsolHSP36 and/or cC1. However, further evaluation is needed to identify the most suitable combination of these proteins. Given the homology between Tsol-p27 and the *E. granulosus* protein P-29, there will most likely be cross-reactivity between those two proteins in patients who are infected with both *T. solium* and *E. granulosus.* However, since the clinical manifestations of NCC and Echinococcosis differ, it should not be difficult to distinguish between the two diseases in order to make a correct diagnosis.

## Figures and Tables

**Table 1:** Identified immunoreactive proteins recognized by NCC-positive human sera (BLAST search performed with an *E*-value cut-off of <10^−5^).

Spot No.	Protein identity	GeneBank accession/organism	Amino acid sequence	M.W. (kDa)	pI
1	Tropomyosin	ABN14948.1/*T. asiatica*	(K)AINLENQLK(E)	32.2	4.7
			(K)NIQTEVDTVQESLQEAISK(L)		
			(R)ATNAEAEVAAMTR(R)		
			(R)LLEEDFEQSSGR(L)		
			(R)MAQLEEQVK(E)		
			(K)YIAEDAER(K)		
			(R)LAVTEVDLER(A)		
			(K)IVELEEELR(I)		
			(K)SLEVSEQESLQR(E)		
			(R)EESYEETIR(D)		
			(K)LQNEVDRLEDELLSEK(E)		
2	Enolase	ACX56268/*T. asiatica*	(K)LGANAILGVSLAVCK(A)	47	6.2
			(K)LAMQEFMILPTGAK(N)		
			SGETEDSTIADIVVGLR		
			(K)HIADLAGNK(N)		
			(K)ACNALLLK(V)		
			(K)YNQLLR(I)		
			(K)VNQIGSVTESIK(A)		
			(R)AAVPSGASTGVHEAVELR(D)		
			(K)IGMDVASSEFYQDGK(Y)		
			LAEIYLEMLSK		
			IEEELGSK		
3	Antigen cC1	AAD34598.1/*T. solium*	NLTPSTLSQVVNPGLAETDAK	37.9	5.7
			EALLLALAGQADEPQAMQLK		
			SFLQLNATNEAYNR		
			YAELYGETLEAAIK		
			ADTDLGSIK		
			DAYPSISSK		
			FALLLIQSPWQVMAEALYDAMK		
			VLNEIIAGCSK		
			SLVHLYAPNGEK		
			TLHDALTSELSGK		
			GDTSGDYEALCLK		
			ASLFAELLHFAMR		
			AFEEVSGGETLDDAIK		
			ELYACGEGRPGTAESR		
4	Actin	AAA30092.1/*T. solium*	(R)VAPEEHPVLLTEAPLNPK(A)	41.7	5.3
			(R)GYSFTTTAER(E)		
			(K)YPIEHAIVTNWDDMEK(I)		
			(K)QEYDESGPSIVHR(K)		
			(K)LCYVALDFEQEMATAASSSSLEK(S)		
			(K)IWHHTFYNELR(V)		
			(K)EITSLAPSTMK(I)		
			(K)IVAPPER(K)		
			(K)AGFAGDDAPR(A)		
			(K)DSYVGDEAQSK(R)		
5	Heat shock Protein p36	Q7YZT0/*T. solium*	(R)NLFSLEPFTAMDNAFESVMK	35.5	5.7
			(R)EFHPELEYTQPGELDFLK(D)		
			(K)SVACGDAAMSESVGR(S)		
			(K)GLAIQPSEVQER(Q)		
			(R)SIPLPPSVDR(N)		
			(K)VYVHGVTGK(E)		
			(K)EMSAIQPR(E)		
			(K)VYFNVK(N)		
6	Tsol-p27	AEF14021.1/*T. solium*	(R)TSDLIHEIDQMK(A)	27	5.0
			(K)IITATEEFVDINIASK(V)		
			(K)LGTALEQVASQSEK(A)		
			(K)NFLNTTLSEAQK(A)		
			(K)EFDGLSVQLLDLIR(A)		
			(K)NYYEACAK(E)		

## HIGHLIGHTS

- Six antigenic proteins were found in *Taenia solium* studied in Mozambique.
- One of these, Tsol-p27, has previously been described as a diagnostic antigen.
- Tsol-p27 was recognized by sera from NCC-positive humans in Mozambique.
